# Power Batteries Health Monitoring: A Magnetic Imaging Method Based on Magnetoelectric Sensors

**DOI:** 10.3390/ma15051980

**Published:** 2022-03-07

**Authors:** Rui Chen, Jie Jiao, Ziyun Chen, Yuhang Wang, Tingyu Deng, Wenning Di, Shunliang Zhu, Mingguang Gong, Li Lu, Xianyu Xie, Haosu Luo

**Affiliations:** 1 Shanghai Institute of Ceramics, Chinese Academy of Sciences, Shanghai 201800, China; chenrui@student.sic.ac.cn (R.C.); jiejiao@mail.sic.ac.cn (J.J.); chenzy_james@sjtu.edu.cn (Z.C.); wangyuhang1007@163.com (Y.W.); linabelldeng@163.com (T.D.); dwn@mail.sic.ac.cn (W.D.); 2 Center of Materials Science and Optoelectronics Engineering, University of Chinese Academy of Sciences, Beijing 100049, China; 3 Department of Instrument Science and Engineering, Shanghai Jiao Tong University, Shanghai 200240, China; 4 Shanghai Motor Vehicle Inspection Certification & Tech Innovation Center Co., Ltd., Shanghai 201805, China; shunliangz@smvic.com.cn (S.Z.); mingguangg@smvic.com.cn (M.G.)

**Keywords:** magnetic imaging technique, power batteries, magnetoelectric sensor array, nondestructive testing

## Abstract

With the popularity of electric vehicles, the ever-increasing demand for high-capacity batteries highlights the need for monitoring the health status of batteries. In this article, we proposed a magnetic imaging technique (MIT) to investigate the health status of power batteries nondestructively. This technique is based on a magnetic sensor array, which consists of a 16-channel high-performance magnetoelectric sensor, and the noise equivalent magnetic induction (NEB) of each channel reaches 3–5 pT/Hz^1/2^@10 Hz. The distribution of the magnetic field is imaged by scanning the magnetic field variation of different positions on the surface. Therefore, the areas of magnetic anomalies are identified by distinguishing different magnetic field abnormal results. and it may be possible to classify the battery failure, so as to put forward suggestions on the use of the battery. This magnetic imaging method expands the application field of this high-performance magnetoelectric sensor and contributes to the battery’s safety monitoring. Meanwhile, it may also act as an important role in other nondestructive testing fields.

## 1. Introduction

With the extensive application of electric vehicles (EVs) in the world, power batteries, as a core component, determine the power performance and mileage of the car. Li-ion batteries are the most successful energy storage devices due to their high energy density. However, the high energy density of these batteries leads to increasingly prominent safety problems [1,2,3]. Nowadays, numerous spontaneous combustion and fire accidents of EV have occurred successively, which causes great concern in terms of the reliability of EV batteries. However, there are few detection methods for the safety of electric vehicle batteries. The most common application method is the current detection technology based on the V–I output curve of the battery pack. This analysis method cannot directly and accurately reflect the defect location after the battery pack is connected in series or in parallel [4], which is inefficient for batch inspection of batteries. Therefore, it is urgent to develop a nondestructive technique (NDT) to monitor the working state of power batteries in production, transportation, operation, and other processes. Furthermore, it is important to find and replace defective batteries in time, in order to reduce EV accidents.

Some NDTs have been previously reported. Finegan et al. [5] reported a temperature monitoring method to track the evolution of internal structural damage and thermal behavior of 18650 NMC cells. This method helps in the design and safety of lithium-ion batteries. Hsieh et al. [6] demonstrated an electrochemical–acoustic time-of-flight experiment to measure the state of charge and health by discussing the density and modulus changes. Siegel et al. [7] showed neutron radiography used in situ for quantification of the lithium concentration, which further monitored the electrode swelling during the charging process. However, these methods still have some limitations in detection efficiency, system resolution, and structure analysis of batteries.

The magnetic imaging technique (MIT) is the most commonly used in various electromagnetic nondestructive evaluation methods [8,9,10,11]. The samples are stimulated through the electromagnetic induction principle, and the state of the samples is assessed according to the distribution of magnetic flux density. On account of different working principles, various kinds of magnetic sensors are employed to image the magnetic field, such as the Hall sensor, the magnetoresistive sensor, and superconducting quantum interference devices (SQUIDs). The Hall sensor is widely used for detecting the magnetic field and the imaging system based on the Hall sensor array can be employed to estimate the crack size and test the shape of ferromagnetic materials [12,13]; however, this method is limited by the sensitivity of the Hall sensor. the Magnetoresistive sensor is manufactured by thin-film technology, which allows them to be highly integrated. Brauchle et al. reported an anisotropic magnetoresistive (AMR) sensor to map the magnetic field and achieve the detection of 227 mA⋅cm^−2^ current density [14]. The TMR sensor also plays an important role in battery magnetic imaging. Researchers inversed the conductivity distribution in batteries according to the test results of magnetic field distribution, which furthered the identification of defect locations and short-circuit current intensity [15,16,17]. Nowadays, the resolution of the TMR sensor can reach several pT at the excitation frequency [18]. However, the preparation process is relatively complex. Compared with the TMR sensor, the SQUIDs work based on the Josephson effect and have been widely used in weak magnetic field detection because of their advantages, such as high sensitivity, large dynamic range, broad frequency bandwidth, and so on. Currently, the magnetic imaging system based on SQUIDs is developed for defects inspection and medical diagnosis [19,20,21]. Nevertheless, SQUIDs require a low temperature to be maintained during operation, which causes the imaging system to be extremely complicated and expensive.

Recently, a new type of magnetoelectric (ME) sensor has drawn much attention. It works by the coupling principle of “magneto–mechano–electric”. The mechanical strain of piezomagnetic materials is induced by an external magnetic field, which simultaneously generates the electric charge of piezoelectric materials by the interface coupling, and thus the signal conversion between magnetic and charge is achieved. Due to the characteristics of high sensitivity, easy fabrication, room-temperature operation, low cost, small size, vector detection, and wide bandwidth (mHz to several tens of kHz), the ME sensor is widely used in weak magnetic signal detection, energy harvesting, antennas communication system, and other fields [22,23,24,25,26]. In this study, we design a magnetic imaging apparatus with a 16-channel sensor array for monitoring the health of power batteries. Since the diversity of current distribution exists during the charging process for healthy and damaged batteries, the magnetic field distribution is also different. Therefore, the distribution diagram of the magnetic field is obtained by scanning the sample surface at the charging moment. Through comparing differences between healthy and faulty batteries, the location of the abnormal magnetic field is identified. We introduce the application of this high-performance sensor in battery health monitoring for the first time. In addition, several kinds of magnetic field anomaly distribution of the damage battery are provided, which may help to determine the type of damage to the battery. Furthermore, this magnetic imaging method may also be beneficial to other NDT fields.

## 2. Experimental

### 2.1. Structural Design of ME Sensor

**Figure 1:**
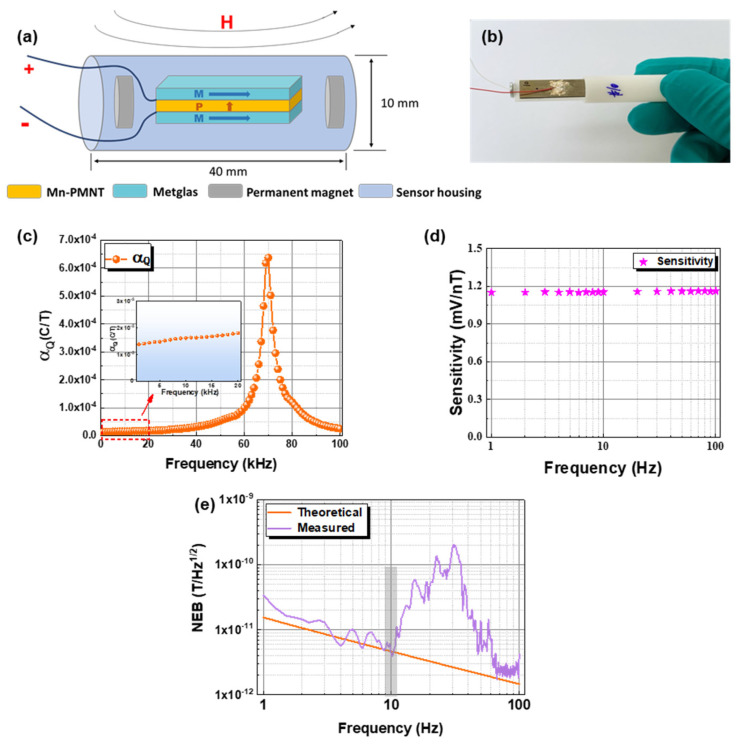
(**a**) Schematic diagram of ME sensor, (**b**) photo of the ME sensor, (**c**) frequency dependence of magnetoelectric coefficient, (**d**) sensitivity of the ME sensor, and (**e**) theoretical and measured NEB values of the ME sensor.

As shown in Figure 1a, the Mn-PMNT piece is made of (110)-oriented Mn-0.71Pb(Mg_1/3_Nb_2/3_)O_3_-0.29PbTiO_3_ single crystal with the dimensions of 14[001]^L^ × 6[−110]^W^ × 0.5[110]^T^ mm^3^ (L: length, W: width, T: thickness), and polarized alone (110) direction. For the piezomagnetic material, Metglas (2605SA1) has a high piezomagnetic coefficient (~4 ppm/Oe), which can be used as the piezomagnetic phase in magnetoelectric materials. The Metglas foils were cut into 30 mm × 8 mm × 25 µm, then four Metglas layers were bonded together by epoxy resin (West System 105/206). Finally, four Metglas foils were bonded to the top and bottom surfaces of the Mn-PMNT piece by using epoxy adhesive. A pair of permanent magnets are used to apply the static magnetic field to ensure the Metglas possess the maximum piezomagnetic coefficient [27,28]. Finally, the size of each encapsulated sensor unit is 10 mm (diameter) × 40 mm (length), as shown in Figure 1b. The change of external magnetic field causes the strain of the Metglas foils, and further generates the Mn-PMNT single crystal produce charge. For the single ME sensor, the frequency dependence of magnetoelectric charge coefficient (α_Q_) is displayed in Figure 1c, α_Q_ is increasing with the frequency rising, and reaching the maximum value (~6.4 × 10^−4^ C/T) around 70 kHz. The charge signal was amplified by circuit, and the sensitivity of the final ME sensor is given in Figure 1d, and the response value is about to 1.15 mV/nT. Figure 1e demonstrates the theoretical and measured noise equivalent magnetic induction (NEB) of the ME sensor, and shows good consistency, although some abnormal peaks still appear around 30 Hz, possibly due to vibrational noise in the environment [29]. The limit of detection (LOD) of ME sensor reaches 5 pT/Hz^1/2^@10 Hz, which exhibits a good weak magnetic detection ability.

**Figure 2:**
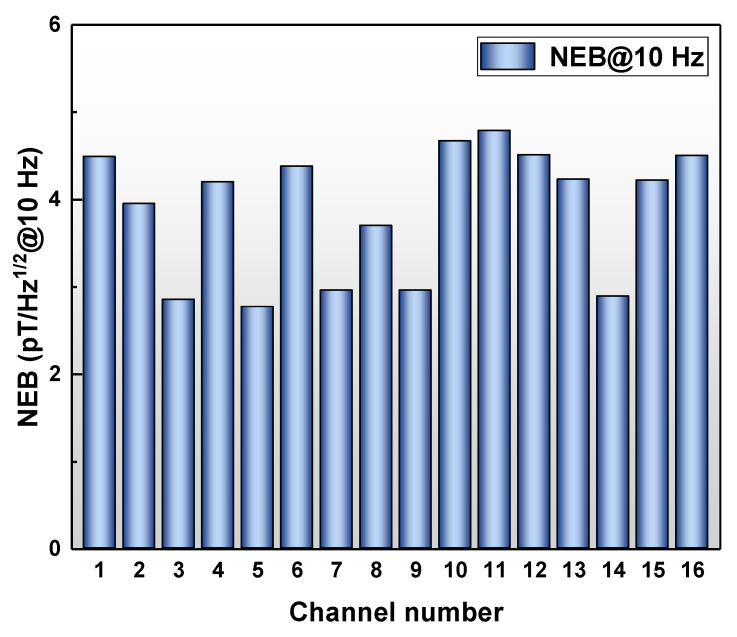
Theoretical NEB@10 Hz of 16 channels.

For the preparation of the ME sensor array, the characteristic parameters of the ME sensor, including capacitance (*C_P_*), dielectric loss (tan*δ*), and magnetoelectric charge coefficient (*α_Q_*), are listed in Table 1. The theoretical NEB value is dominated by dielectric loss noise in the low-frequency range, which can be calculated with the following Equation (1) [22,29,30]:

(1) $$NEB=\frac{\sqrt{\frac{4k_{B}TC_{P}\tan\delta }{2\pi f}}}{\alpha_{Q}}$$

where *f* is the frequency in Hz, *k_B_* is the Boltzmann’s constant (1.38 × 10^−23^ J⋅K^−1^), *T* is the temperature in K, and *α_Q_* is magnetoelectric charge coefficient in C/T. The calculated results are presented in Figure 2 and NEB values of 16 channels are distributed in 3–5 pT/Hz^1/2^@10 Hz, which means that they can distinguish weaker magnetic signals, and may contribute to the NDT field.

### 2.2. Testing Process

**Figure 3:**
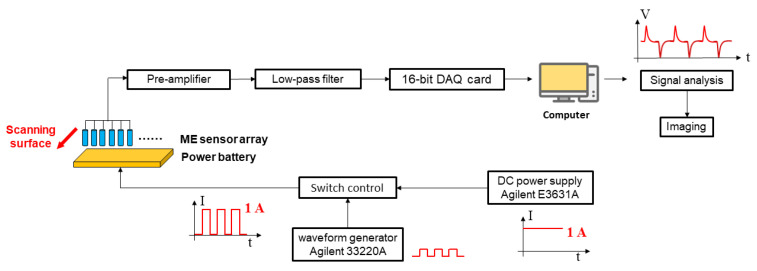
Diagram of the experiment setup.

The diagram of the experimental setup is demonstrated in Figure 3. Since the leakage current of piezoelectric materials exists in extremely low frequency or DC situations [31,32], the ME sensor only responds to changing magnetic signals. A periodic rectangular wave-pulsed current is used to produce the momentary change in the magnetic field. The batteries used in this work are multilayer soft packing batteries, the operation range of voltage is 2.5 V~4.2 V, and the capacity is 50 Ah. The DC power supply (Agilent E3631A, Murrieta, CA, USA) provides a constant current with 1 A, which is controlled by a rectangular pulse signal (frequency = 0.25 Hz, duty cycle = 50%) coming from the wave generator (Agilent 33220A, Murrieta, CA, USA); the final rectangular pulse current with 1 A is formed. When this current charges the power batteries, the variation of the magnetic field is measured through the 16-channel ME sensor array by scanning the surface of the batteries.

The output signal of the ME sensor array is amplified by the voltage amplifier (the magnification is 100 mV/mV) and filtering by a low-pass filter (remove noise signal above 10 Hz), then connected to a 16-bit DAQ card (National Instruments USB-6210, Austin, TX, USA) with a sampling rate of 250 kS/s, and the further output result is recorded and analyzed by LabView SignalExpress 2015 (Austin, TX, USA). Finally, the recorded data is employed to image the magnetic field. 

**Figure 4:**
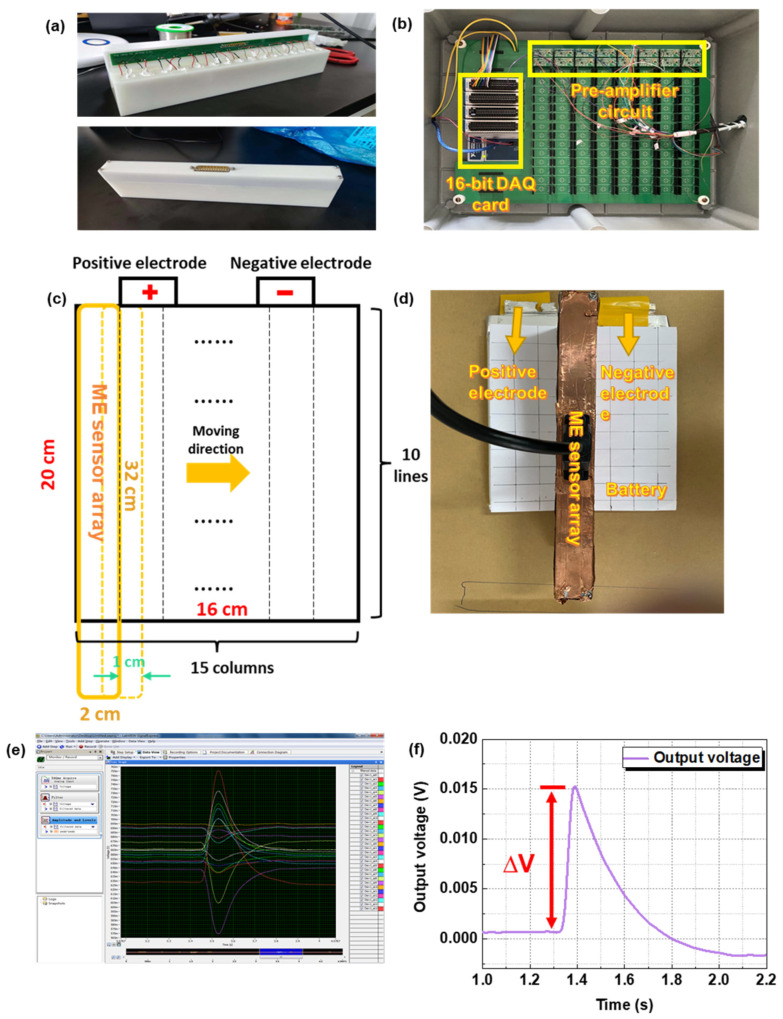
Picture of (**a**) the encapsulated array sensor, (**b**) pre-amplifier and 16-bit DAQ card, (**c**) schematic diagram of magnetic imaging scanning process, (**d**) placement of sensor array and batteries sample, (**e**) output waveform on Signal Express software interface, and (**f**) typical single channel output voltage.

A picture of the ME sensor array is presented in Figure 4a. The 16 channels’ outputs are linked to a printed circuit board (PCB) for signal transmission. The pre-amplifier and 16-bit DAQ card are exhibited in Figure 4b. The sensor array is vertically placed for measuring the Z-axis magnetic field B_Z_. A schematic diagram of the magnetic imaging scanning process is shown in Figure 4c,d. The size of each test point is 2 cm × 2 cm, and 16 sensors are positioned in a linear arrangement to form a sensor array (2 cm × 32 cm). The size of the batteries is 16 cm × 20 cm. We laterally move the ME sensor array step by step (1 cm for each time) and keep the batteries still. Thus, the 15 columns of data are obtained in transverse direction and 10 lines of data in longitudinal direction. A total of 150 data points (15 columns, 10 lines) are filled by the linear interpolation method to improve the imaging quality. Finally, 9600 (120 columns, 80 lines) points are used to create the imaging result. The final output waveforms on the software and a typical output voltage are given in Figure 4e,f (under an external magnetic field of 10 nT). The degree of the signal bulge (∆V) of each channel represents the magnetic field intensity for different positions. Thus, the specific magnetic field can be calculated according to the following Equation (2):

(2) $$B=\frac{\Delta V}{R^{*}}$$

where *B* is the magnetic field intensity of different positions, ∆*V* is output voltage change, and R* is the responsivity of each channel of the sensor array.

## 3. Results and Discussion

**Figure 5:**
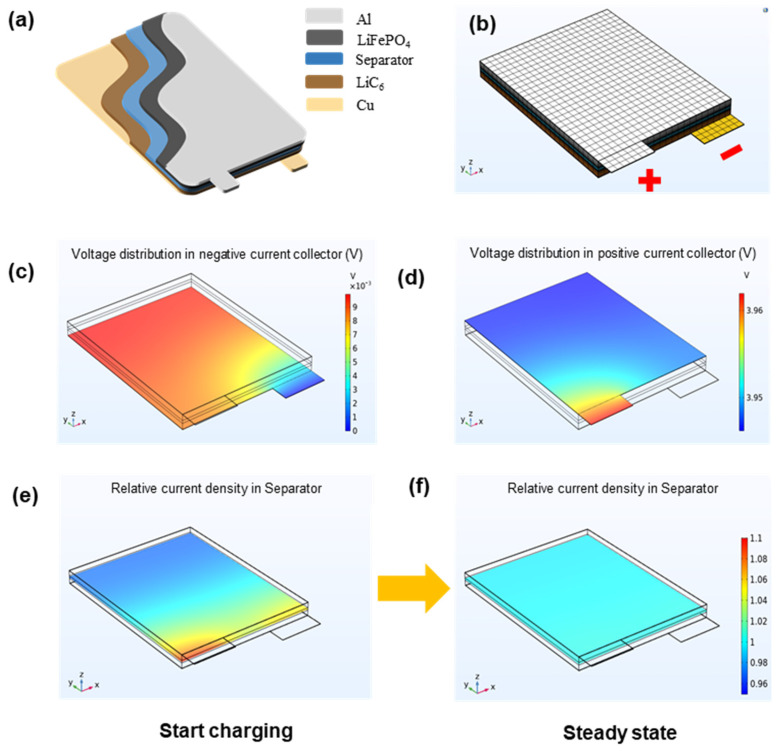
(**a**) Internal structure diagram of power batteries, (**b**) mesh generation of the model in FEA software, voltage distribution in (**c**) negative current collector and (**d**) positive current collector at t = 0 s, relative current density with time variation: (**e**) start charging; (**f**) steady state.

According to the law of electromagnetic induction, the distribution of the magnetic field depends on the distribution of current. Thus, the relative current density (ratio of instantaneous current to average current) inside the batteries at the charging moment is analyzed firstly. Figure 5a demonstrates the internal structure diagram of the power batteries, which is composed of the positive current collector with Al, a negative current collector with Cu, positive electrode LiFePO_4_, negative electrode LiC_6_, and separator. The finite element analysis (FEA) software-COMSOL Multiphysics 5.6 (2021, Sweden) is used to analyze the internal voltage and current distribution at the charge moment. The mesh generation of the batteries model in FEA software is clarified in Figure 5b. For the charging condition of 4 V 1 A, the voltage distribution of negative and positive current collectors at the moment of charging is exhibited in Figure 5c,d, and it can be clearly found that the voltage in the current collector is inhomogeneous; the voltage near the ground terminal is lower than the average level (Figure 5c), while the voltage near the input terminal is also higher than the average level (Figure 5d). Hence, this inhomogeneity of voltage will cause an uneven current distribution in the charging process. The result of relative current density with time-variation is described in Figure 5e,f. At the beginning of charging, it is obvious that the relative current density near the electrode tab is higher than other regions, which may lead to some abnormal magnetic field regions on the surface of batteries. However, as the charging time increases, the relative current density tends to be uniform at steady state in the whole battery.

**Figure 6:**
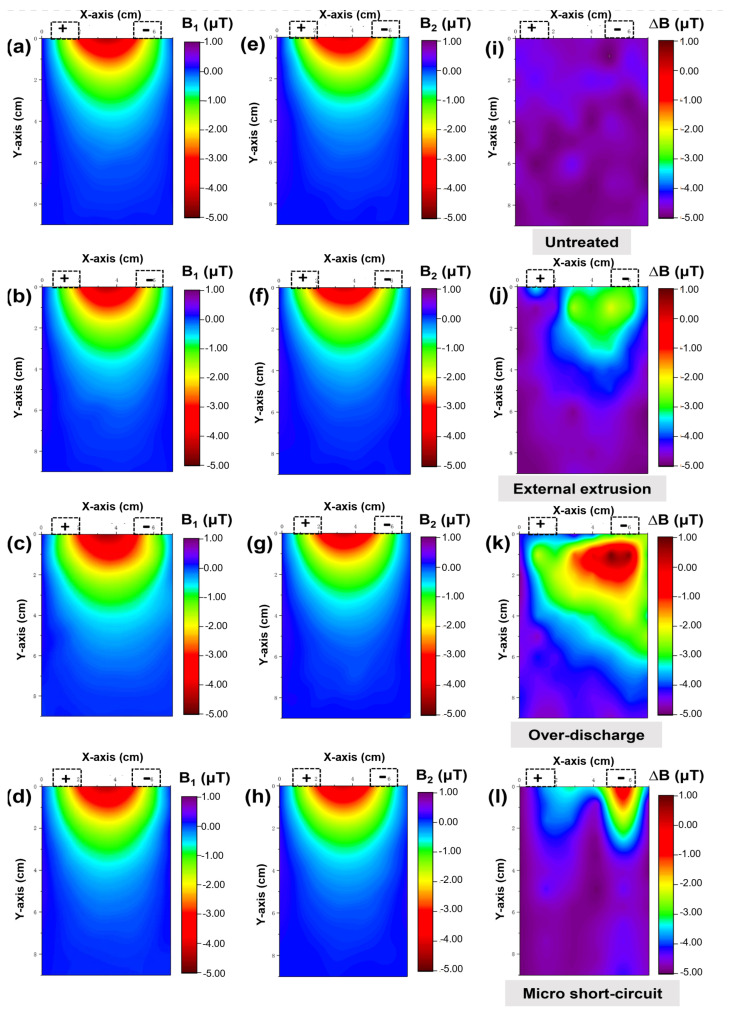
(**a**–**d**): Magnetic field distribution of healthy power batteries before treatment (B_1_); (**e**–**h**): magnetic field distribution of power batteries after different treatments (B_2_); and magnetic field variation (∆B) of different samples: (**i**) untreated, (**j**) external extrusion, (**k**) over-discharge, and (**l**) micro short circuit.

This section discusses the magnetic imaging results of three kinds of faulty situations of power batteries, including external extrusion, over-discharge, and micro short circuit, and in addition, the untreated sample is used as a control group. the magnetic imaging results of healthy batteries (B_1_) are presented in Figure 6a–d. It is easy to notice that the magnetic field near the top central area is much higher than other regions, and the reason can be explained by the higher current density in the batteries tab, which is simulated in Figure 5e,f. The figures show the input and the output current generated and the superimposed magnetic field used to obtain these results. The magnetic field near the batteries tab is so high that it covers the defect location; hence, we measured the magnetic field distribution after the artificial damage treatment (B_2_) and subtracted the B_1_ value to compare the magnetic field anomaly (∆B = B_2_ – B_1_). The magnetic field after artificial damage treatment is imaged in Figure 6e–h. These figures display a slightly larger gap than before. The values of ∆B are given in Figure 6i–l. Figure 6i shows the control group; the ∆B value of the untreated sample is close to 0. For the sample after the external extrusion, the top magnetic field varies little (about 0.2 µT). This situation could be caused by the small change in layer spacing in the pouch cell, which leads to the result of the magnetic field superposition changes. The ∆B value of the over-discharge batteries exhibits an obvious magnetic anomaly in Figure 6k, and the maximum reached is 0.6 µT. For the over-discharge process, the copper foil on the negative collector is oxidized and deposited on the surface [33,34,35], leading to the uneven current density and causing magnetic field anomaly eventually. For the last condition, a micro short-circuit environment is established by connecting the external resistance with 500 Ω. As shown in Figure 6l, the magnetic field discrepancy is mainly existing near the two electrode tabs, and the reason may come from an additional external leakage current.

## 4. Conclusions

A novel magnetic imaging technology based on a magnetoelectric sensor is presented to characterize the healthy state of batteries, which provided relatively accurate and direct abnormal magnetic field results on the battery surface. When battery samples are damaged during transportation or operation, the internal structure or material changes, which affects the current density distribution and creates magnetic field anomalies on the surface. These abnormal magnetic field results help us analyze the problems of the battery. In this paper, we first fabricated a high-performance magnetic sensor array with a low-noise level (NEB: 3–5 pT/Hz^1/2^@1 Hz). Then, we developed a test method by scanning the surface magnetic field on the battery under a periodic rectangular wave pulse current excitation. Finally, several kinds of battery damage modes, including external extrusion, over-discharge, and micro short circuit, were characterized by this magnetic imaging method. These different kinds of magnetic field distribution abnormalities may help us later identify additional damage modes by accumulating more test results.

In the future, the size of the magnetoelectric sensor can be reduced to improve the integration and further enhance the spatial resolution of the magnetic imaging system. Meanwhile, the scanning speed of the sensor array can be increased to improve the working efficiency of the magnetic imaging system. In addition, the test results of more batteries with different packaging structures need to be provided. A modified version of this magnetic imaging system is already under development. Finally, this magnetic imaging method may provide valuable information for the damage modes of batteries, which will contribute to monitoring the status of batteries during the process of use. In addition, this technology can also be extended to other non-destructive testing fields.

## Figures and Tables

**Table 1:** Characteristic parameters of the ME sensor.

Channel Number	*C_P_* (nF) @1 kHz	tan*δ* (%) @1 kHz	*α_Q_* (×10^−8^ C/T) @1 kHz
1	2.58	0.50	1310
2	2.07	0.51	1350
3	2.09	0.30	1450
4	2.62	0.47	1370
5	2.20	0.19	1240
6	2.61	0.57	1440
7	2.13	0.34	1500
8	2.78	0.39	1460
9	2.63	0.25	1430
10	2.74	0.47	1260
11	2.78	0.37	1100
12	2.84	0.43	1270
13	2.56	0.38	1210
14	2.46	0.28	1500
15	2.56	0.43	1290
16	2.30	0.58	1330

## Data Availability

Not applicable.
